# For-me-ness: a critical assessment of the phenomenal mineness thesis

**DOI:** 10.1007/s10339-026-01383-z

**Published:** 2026-08-04

**Authors:** Roberto Sá Pereira

**Affiliations:** Department of Philosophy, Universidade Federal do Rio de Janeiro, Rio de Janeiro, Brazil

**Keywords:** For-me-ness, Self-consciousness, Self-awareness

## Abstract

This paper examines the claim that the phenomenal “for-me-ness” is the core feature of every conscious experience. Any conscious episode is intrinsically given as mine, and this mineness, while non-reflective and non-cognitive, belongs to the personal and phenomenal level. The compatibility checks for the for-me-ness thesis comes from cases such as depersonalization, schizophrenia, infancy and autism. I argue that this view lacks evidential support beyond the phenomenological descriptions it was built to interpret and that these phenomena receive better explanations that do not require positing for-me-ness.

## Introduction

Over the last three decades, phenomenology has returned to mainstream philosophy of mind and philosophy of cognitive science. Thinkers such as Dan Zahavi (2005, 2014) and Shaun Gallagher (2000, 2005, 2012), along with Michelle Montague (2009, 2011, 2013, 2016, 2018), Mark Rowlands (2013), Robert Howell (2019), Manfred Frank (2022), and Uriah Kriegel (2003), Evan Thompson (2007, 2011), Matthew Ratcliffe (2008), Louis Sass (2003, 2014), Josef Parnas (2002, 2003), Thomas Fuchs (2005, 2013), Michel Bitbol (2012), and Natalie Depraz (2017), claim that classical phenomenology still offers strong tools for understanding subjectivity, embodiment, and intersubjectivity. This rediscovery builds on a longer tradition in which consciousness is seen as self-manifesting.

ll’s analyses of inner time-consciousness and the stream of experience (1905/1991; 1913/1983) are often read as showing that experience involves a constant, tacit *self-presence*. Sartre (1943/1956) writes that “consciousness is a being such that in its being its being is in question in so far as this being implies a being conscious of itself” (p. 130). Merleau-Ponty (1945/2012) stresses the bodily side of this self-presence, describing the lived body *as my body*, the zero-point of orientation and action (pp. 90–93).

The first tenet of this “rediscovery” is that “inner perception” is not an internal act of observation directed at one’s own mental states. It is not a second-order monitoring event. It is the mode in which each conscious state is given from within its own point of view (Zahavi 2014, pp. 26–33). It marks the built-in self-givenness of experience. Every experiential token presents itself pre-reflectively as lived and as mine.

This minimal self-appearance is neither an extra act nor an introspective mechanism (Zahavi 1999, 2005). In Zahavi’s neo-phenomenological view, it amounts to the thesis of pre-reflective self-awareness, a structural and non-objectifying self-presence built into first-person experience. It has a first-personal character, a “for-me-ness” or “mineness,” said to be present whenever anything is consciously experienced. Zahavi writes that “whenever I undergo an experience, there is a minimal sense in which it is given as my experience,” and that this pre-reflective self-givenness is “a built-in feature of experiential life” (2005, pp. 15–16).

Gallagher argues that “conscious experience is characterized by a pre-reflective sense of ownership” and that this “is not added on by some further act of reflection but is intrinsic to the experience itself” (2000, p. 15). Thompson similarly speaks of “a primitive, pre-reflective sense of self or mineness that is inseparable from the stream of consciousness” (2007, p. 158), and later stresses that this “pre-reflective sense of mineness is a basic, non-conceptual feature of conscious experience that does not depend on higher-order thought or conceptual self-ascription” (2011, p. 160). Ratcliffe likewise maintains that experience is “always someone’s experience, marked by a distinctive first-person character” (2008, p. 3). Montague also defends the idea that conscious experience involves an inbuilt first-personal, self-referential dimension, treating the subject’s presence as an integral part of consciousness rather than a reflective add-on.

The second tenet of this rediscovery is the rejection of views that treat the self as a construct. The idea is that every conscious experience has an intrinsic, pre-reflective first-personal character that does not come from narrative, introspection, or social processes (Zahavi 1999, pp. 15–20; 2005, pp. 105–112; 2014, pp. 26–33; 2018, pp. 19–24). This contrasts with Dan Dennett’s view that the self is a narrative fiction built by interpretive mechanisms (Dennett 1991, pp. 81–95). It also differs from Antonio Damasio’s claim that “the self comes to the brain” through mapping layers and homeostatic processes rather than through a built-in first-person structure (Damasio 1999, pp. 153–168). Finally, it diverges from Paul Ricoeur’s view, which places selfhood in narrative and hermeneutic mediation, treating the self as an interpretive construction rather than as a structural feature of lived experience (Ricoeur 1992, pp. 114–121).

The third tenet of the neo-phenomenological movement is the claim that Husserl’s classical transcendental project must give way to a naturalized but non-reductive framework aligned with empirical cognitive science. Husserl held that transcendental subjectivity serves as the ultimate ground of meaning and cognition, insisting that phenomenology identifies “the absolute source of all legitimacy of knowledge” (Husserl 1913/1983, p. 84) and that the transcendental ego is “the carrier of all intentional life” (Husserl 1931/2012, p. 111). Neo-phenomenologists argue that this insulation from the sciences is no longer viable. Zahavi rejects strong transcendental isolation and maintains that phenomenology must remain empirically responsible while preserving the irreducibility of lived experience (Zahavi 2017, p. 42). Gallagher likewise holds that analyses of embodiment and agency should function as constraints on cognitive science rather than as pre-scientific foundations (Gallagher 2020, p. 15).

This shift appears across several authors. Depraz defends a naturalized phenomenology tied to experimental practice (Depraz, 1999, p. 170). Thompson advances an enactive model compatible with phenomenology while rejecting reductive physicalism (Thompson 2007, p. 45). Ratcliffe integrates phenomenological accounts of affect with empirical psychiatry (Ratcliffe 2008, p. 12). Fuchs promotes an ecological-phenomenological account of embodiment continuous with dynamical neuroscience (Fuchs 2018, p. 33). De Jaegher embeds phenomenological analyses of interaction within a naturalistic model of participatory sense-making (De Jaegher 2013, p. 23).

I do not deny that experience has a perspectival structure or that subjects normally report their experiences as their own. Perceptual and bodily states present the world from a here-centered point of view, and ordinary first-person claims like ‘I feel pain’ or ‘I see the tree over there’ treat experiences as the subject’s. I also do not deny that we can make sense of pre-reflexive self-consciousness. My target is the stronger claim that perspectival character amounts to a primitive, *phenomenal* mineness or for-me-ness that is built into what it is like to have any experience at all, prior to and independent of reflection or self-ascription. Zahavi writes that ‘there is an experiential difference between my experience and your experience, a difference that is not simply a difference in qualitative character, but a difference in first-personal givenness’ (Zahavi 2014, p. 23). Even if Zahavi rejects this construal (he probably would), the present argument shows that the empirical and explanatory roles often attributed to phenomenal perspectivity do not require construing it as a primitive experiential structure.

The argument of this paper presupposes a general view of consciousness that should be made explicit at the outset. Some authors distinguish for-me-ness from ownership or mineness, reserving the former for the privacy or first-personal availability of experience (Guillot 2017). My argument targets the stronger thesis that for-me-ness, in this sense, amounts to a phenomenally manifest structure that explains ownership and self-presence. Weaker notions of mere for-someone-ness are not in dispute and do not support the minimal self-hypothesis as an explanatory claim.

Having clarified the target of my critique, I reject the claim that consciousness constitutively involves a built-in phenomenal selfhood or an intrinsic dimension of for-me-ness. At the same time, I do not deny the reality of conscious experience, its perspectival character, or its first-person accessibility. I assume, rather, that conscious states are contentful and world-directed, with accuracy conditions: they represent objects, properties, bodily conditions, and events in ways that can be accurate or inaccurate.

It is important to emphasize, however, that I remain neutral on further metaphysical questions concerning the ultimate nature of phenomenal qualities. The argument of this paper therefore does not depend on any reductive thesis about qualia, but only on the methodological requirement that appeals to phenomenal structure must earn their place by doing genuine explanatory work. Accordingly, I adopt a broadly naturalistic orientation, on which theories of consciousness are assessed by their capacity to account for empirical patterns, explanatory constraints, and systematic dissociations, rather than by appeal to primitive experiential postulates.

This stance is incompatible with neo-phenomenological views that treat pre-reflective self-givenness as a basic and irreducible feature of experience. At the same time, it does not commit me to higher-order theories, eliminativism, or the denial of phenomenal consciousness. The alternative developed in this paper should therefore be understood as a non-phenomenological, empirically constrained account of conscious experience—one that dispenses with primitive for-me-ness while preserving the explananda of perspectival content, bodily anchoring, and first-person authority.

Defenders of the phenomenal for-me-ness in the classical phenomenological tradition often rely on pre-theoretical descriptions that already assume subjective ownership. Husserl’s claims that the ego is “always already there” (1913/1983, p. 105), that every experience bears an “internal index” pointing back to an ego (1931/1960, p. 84), and that no lived experience is anything but “my own” (1931/1960, p. 68) exemplify this pattern. These descriptions render anonymous experience conceptually unavailable from the outset, turning for-me-ness into a methodological assumption rather than a phenomenological result that demands independent support.

Yet in the naturalistic phase of neo-phenomenology, Zahavi and Gallagher appeal to depersonalization, schizophrenia, infancy, and autism as independent empirical evidence for the phenomenal for-me-ness. Zahavi claims that depersonalization is ‘intelligible only if’ normal experience carries mineness, and Gallagher attributes to early infancy a built-in sense of ownership. Yet, it should be noted that Zahavi treats these pathological cases as compatibility checks rather than direct positive evidence.

The key point of this paper is that contemporary cognitive science and neuroscience offer far more precise accounts of these cases through multisensory integration, predictive processing, and disruptions in self-modelling. My argument against phenomenal for-me-ness (mineness) is simple. The posit adds no explanatory value and becomes an unnecessary theoretical commitment. The issue is therefore not whether alternative explanations are possible, but whether the phenomenal for-me-ness hypothesis yields explanatory benefits that justify its additional theoretical commitments when compared with empirically grounded alternatives.

In what follows I offer an alternative view that keeps the perspectival character of experience but does not posit a primitive, phenomenally present for-me-ness. On this view, conscious states are world-directed presentations shaped by non-conscious forms of what I call “egocentric registration.” Information is encoded in an egocentric frame centered on the subject’s body, but the subject does not need to be phenomenally aware of this centering as mineness. What phenomenologists call pre-reflective for-me-ness can, I suggest, can be explained away by this non-conscious egocentric registration together with ordinary, concept-involving acts of self-ascription.

A preliminary methodological clarification is in order. The neo-phenomenological for-me-ness thesis is often presented as empirically informed, yet the alleged evidence for it is invariably interpreted through the very framework it is meant to support. This gives rise to a familiar methodological worry: the thesis lacks clear falsifiability conditions, even in a weak sense. The problem is not that for-me-ness cannot be directly measured, since it is characterized as a formal feature of experience, but that it remains unclear what kinds of observations would count against the claim that every conscious state is intrinsically marked by mineness. In particular, if reports of absorption, depersonalization, pain, infancy, and autism are all treated as compatible with the continued presence of for-me-ness, then the thesis accommodates any possible phenomenological data. A position with this degree of flexibility has, at best, a hermeneutic status rather than an explanatory one.

This methodological diagnosis does not presuppose a restrictive Popperian notion of falsifiability. It relies instead on a weaker and widely accepted requirement: that a theory be open, in principle, to revision in light of recalcitrant data, understood in Quine’s sense as information that undermines, weakens, or calls into question a hypothesis or belief.

This concern is made explicit in Sect. For-me-ness as ubiquity, where absorbed experience is examined not as a substantive counterexample but as a test case for irrefutability. The central point is that the same phenomenological descriptions of absorption are equally compatible with the presence or the absence of phenomenal mineness, thereby failing to discriminate between competing accounts.

Finally, the for-me-ness thesis carries implicit empirical commitments. If mineness is a global and necessary feature of consciousness, then disruptions of interoception or affective processing should result in uniform and global alterations in experiential ownership. By contrast, if ownership depends on more local and dissociable mechanisms, we should expect selective disruptions and dissociations—precisely the pattern observed in depersonalization and related conditions.

This paper is divided into the following sections. Section The for‑me‑ness thesis presents the phenomenal for‑me‑ness thesis and distinguishes it from perspectival structure and ordinary self‑ascription. Section For-me-ness as proprioception examines the proposal that for‑me‑ness is grounded in proprioceptive bodily awareness. Section For-me-ness as ubiquity considers the idea that for‑me‑ness is a ubiquitous feature of absorbed experience. Section For-me-ness as pain discusses whether pain provides a paradigm case for intrinsic mineness. Section For-me-ness in DPD turns to depersonalization–derealization disorder and evaluates whether its heterogeneous symptoms support a global for‑me‑ness model. Section For-me-ness in infancy and autism assesses claims from infancy and autism regarding early or atypical forms of mineness. Section Egocentric registration as weak for-me-ness develops an alternative account centered on non‑conscious egocentric registration. Section Objections and replies presents objections and replies to this alternative framework. Section Conclusion presents the concluding remarks.

### The for‑me‑ness thesis

For much of the twentieth century, phenomenological claims about first-person givenness stayed peripheral in analytic philosophy of mind. Dominant views such as logical behaviorism, identity theory, and functionalism either bracketed the issue or treated it as reducible to functional or representational roles. Over the past thirty years, this situation has changed. The rise of consciousness studies and the formulation of the problem of consciousness renewed attention to the subjective character of experience. Growing dissatisfaction with purely third-person, computational, or representational models also encouraged a renewed interest in phenomenology as a framework that can engage productively with cognitive science.

Gallagher and Zahavi exemplify this neo-phenomenological turn, arguing that classical phenomenology is not a historical relic but a framework that informs research on subjectivity, embodiment, and intersubjectivity, and can guide empirical work (Gallagher and Zahavi 2008, p. 23). Montague (2016, pp. 2–3, 41–42) defends a Brentanian view according to which “all conscious awareness in some manner constitutively involves awareness of that very awareness,” so that “awareness of the object and awareness of that very awareness constitute a single mental state.”

Rowland (2013, pp. 111–112) likewise claims that each conscious episode includes “a built-in sense that there is an experience occurring for a subject,” a “pre-reflective consciousness of consciousness itself.” Kriegel (2006, pp. 143–145) develops a same-order self-representation model on which a conscious state “represents itself and its primary object in a single, unified act of awareness,” thereby explaining how it “presents itself as mine.” Thompson (2007, p. 158; 2011, p. 160) speaks of “a primitive, pre-reflective sense of self or mineness,” and Ratcliffe (2008, p. 3) describes experience as “always someone’s experience, marked by a distinctive first-person character.”

Fuchs (2013, p. 247) characterizes basic selfhood as “a pre-reflective self-presence, a minimal for-me-ness.” Bitbol (2012, p. 314) writes that experience “is always given in the first person, with an intrinsic for-me-ness,” and Depraz (2017, p. 177) speaks of “a structural feature of lived experience, a tacit for-me-ness that comes before and grounds any reflective self-description.” These authors reject reductive third-person accounts (Dennett 1991) and eliminativist models (Metzinger 2003), insisting that a serious science of mind must rely on disciplined first-person inquiry whose central datum is pre-reflective mineness. Zahavi formulates the view explicitly: whenever a subject has an experience, “there is a minimal sense in which it is given as my experience,” a “built-in feature of experiential life” (Zahavi 2005, pp. 15–16). Gallagher similarly claims that “conscious experience is characterized by a pre-reflective sense of ownership” independent of higher-order thought (2000, p. 15; 2005, pp. 173–176), and argues that this embodied mineness is present already in early infancy (Gallagher 2012, p. 12).^1^

To assess the neo-phenomenological defense of for‑me‑ness, we need a clearer map of the terrain. In the literature, at least three distinct ideas are often treated as if they formed a single package: (a) the perspectival or egocentric structure of experience, (b) ordinary practices of self-ascription and first-person discourse, and (c) the stronger claim that there is a primitive, pre-reflective “for‑me‑ness” that is an intrinsic phenomenal feature of every conscious state. I argue that much of the apparent force of the for‑me‑ness thesis comes from an unexamined slide—from (a) and (b) to (c). In this section, I separate these claims and show how my target differs from what even a reductionist-averse, phenomenology-friendly cognitive science must accept.

First, many authors claim that conscious experience is perspectival or egocentrically organized. Perceptual episodes and bodily sensations present a world from a specific position, never from nowhere. Objects appear relative to a here-centered point, as nearer or farther, reachable or not. Bodily states are felt as located in a particular organism. Classical phenomenology captures this by calling the body the “zero-point” of orientation (Husserl) or the “third term” that anchors mind and world (Merleau-Ponty, 1945/2012, pp. 90–93). This point is uncontroversial. My argument does not deny that experience is perspectival or that processing is egocentrically structured.

Second, subjects normally take their experiences to be their own, and this appears in language, thought, and action. We say “I feel pain,” “I see the tree,” “I am thinking about tomorrow,” and “my leg is numb.” First-person pronouns help us mark our experiences as ours and guide social coordination and behavior. In Husserl’s “natural attitude,” we live among enduring persons and bodies, and we place conscious episodes within our autobiographical narratives. Neo-phenomenologists argue that these first-person practices should neither be reduced to third-person descriptions nor dismissed as “user illusions,” but should guide and constrain empirical research (Gallagher and Zahavi 2008; ch. 1, pp. 10–15). 2

A further and often implicit step is the claim that this pre-reflective for-me-ness is *necessary* for consciousness. Neo-phenomenologists usually reject the idea of an “anonymous” conscious state, an experience that presents the world but is in no sense someone’s experience (see Metzinger 2003). They hold that there is always a tacit sense that experiences are mine and that experience is not an anonymous flow but is always given in the first person, with an intrinsic for me ness (Parnas, 2003, p. 218, Bitbol 2012, p. 314, Zahavi 2005, pp. 15–16, Thompson 2007, p. 158, Ratcliffe 2008, p. 3, Sass and Parnas, 2003, p. 429, Fuchs, 2013, p. 247, Depraz 2017, p. 177). They take the idea of a conscious but non-owned or subjectless experience to be incoherent.

I accept that experience is perspectival and egocentrically organized, and that people normally treat and describe their experiences as their own. I question whether we are justified in adding a further claim, namely that there is a primitive experiential mineness that (i) is present in every conscious state, and (ii) is required for consciousness. The next sections argue that the phenomenological evidence for this claim is weak and that the explanatory work tied to for-me-ness can be done by non-conscious egocentric registration plus ordinary, often concept-involving, self-attribution.

### For-me-ness as proprioception

What is the for-me-ness thesis? Gallagher (2003) gives the clearest statement, drawing on Heidegger’s distinction between the ready-to-hand (*Zuhandenheit*) and the present-at-hand (*Vorhandenheit*) (Heidegger, 1927/1996). Gallagher argues that bodily self-awareness is a non-observational, pre-reflective awareness of the body as subject, the lived source of action, not as an object in a perceptual scene. This background somatic proprioception guides your movements and supports your sense of moving or sitting with your legs crossed, without presenting the body under an object-identifying profile. It does not meet the identification constraint or place the body in an egocentric frame. It tracks the body from within in a non-perspectival way.

Before proceeding, it is important to clarify the limited ambition of this section. I do not aim to settle the broader debate between sensation-based and perception-based accounts of bodily awareness, which has been extensively discussed in the literature. The present argument is more local: it asks what bodily awareness must explain in order to support the minimal self or for-me-ness thesis. In particular, I am concerned with whether bodily awareness requires positing a phenomenally manifest structure of mineness, rather than with its correct classification within general theories of perception.

For Gallagher, bodily self-awareness is non-perceptual and pre-reflective. It is awareness of the lived and acting body you move and inhabit, not one more object in the world. By contrast, when you see your arm or touch your skin, your body appears as an object in the same intentional format as other things. You identify it, track it, and locate it in egocentric space as to the left, nearer, or farther. In visual or tactile self-perception, the hand or arm is picked out as this object. Recessive proprioception remains in the background as the for-me-ness of being the one who sees or touches. On this view, perception of your own body rests on non-perceptual bodily self-awareness.

Let us grant, for the sake of argument, that tactile and proprioceptive states are not object-representations, even though much work in cognitive science and philosophy of perception treats perceptual constancies as present in tactile, auditory, and proprioceptive modalities. 3 On this assumption, feeling your legs crossed is not like seeing them crossed in a mirror. In somatic proprioception, you are the subject of the state, not an object in a scene. When you touch something, including your own body, you may represent the touched item as an object, but the self-specifying feedback you receive is not a representation of yourself as one object among others.

Is Gallagher’s view right? We agree with him that proprioception is not perception in the usual sense. It does not represent distal particulars or their attributes. It does not rely on perceptual constancies such as size, shape, or surface orientation. It does not objectify the environment by representing stable, re-identifiable items in a scene.

We concur with Gallagher that proprioception isn’t perception. To be sure, it doesn’t represent external objects or their attributes, nor does it rely on perceptual constancies like size or shape. It also doesn’t objectify the environment by representing stable, identifiable items.^4^ However, proprioception *does represent* the body and its parts, capturing limb positions, joint angles, and muscle stretches. Therefore, these proprioceptive states have accuracy conditions and support body constancy, treating the body as a unified, bounded entity rather than a collection of isolated sensations. In mammals, proprioception integrates with vision and touch, helping guide actions and stabilize spatial orientation. This integration highlights how proprioception underscores the distinction between non-conscious bodily feedback and object-oriented perception.

Does the evidence favor Gallagher’s view or my alternative view? The empirical data—behavioral, neurological, and clinical—better supports my view. Proprioception appears to be a genuine representational capacity, with accuracy conditions and body-level objectification. In contrast, the phenomenological-enactivist perspective treats proprioception as simply skillful bodily engagement, downplaying internal representations. On the other hand, my alternative view argues that proprioceptive states specify bodily properties—like limb position and muscle stretch—that can be accurate or inaccurate, explaining action success, illusions, and pathologies.

Experimental and clinical research on proprioceptive illusions supports the idea that these states have accuracy conditions. For example, vibrating a tendon can create a false sense of limb movement, and altering visual feedback can shift one’s perception of limb position. These shifts are not random but are structured errors about bodily parameters, showing that proprioception represents the body in specific, measurable ways. Gallagher’s enactivist view can describe these cases as changes in bodily skill, but it doesn’t explain why these errors align so closely with specific bodily parameters. My alternative view, however, predicts that proprioceptive states carry contents that can be correct or incorrect, reflecting how the body is actually positioned.

The same applies to body constancy and body-schema effects. Coordinated movement, stable posture, and precise whole-body actions in humans and vertebrates indicate a system that perceives the body as a unified, bounded whole with defined parts and spatial relationships. Disorders like neglect or limb size distortion highlight specific disruptions in body representation. Studies on tool use further show that tools can integrate into this body map, extending reach and altering perceived space. This supports the idea that proprioception forms a stable map of the body’s shape and limits.

Debates over bodily ownership typically focus on body image rather than body schema, and I do not deny this distinction. However, body image representations themselves depend on, and are constrained by, non-conscious egocentric and proprioceptive structures implemented at the level of the body schema. If bodily ownership or mineness were a primitive phenomenal feature, it should persist independently of variations and disruptions in these underlying mechanisms. The fact that ownership varies systematically with changes in egocentric and proprioceptive processing therefore supports a deflationary account on which ownership is constructed rather than basic.

It is important to clarify the role that the discussion of intentionalism plays in the present argument. The aim is not to claim that representational or intentional structure by itself yields bodily ownership, nor that resolving the intentionalism–sensation debate would settle the minimal self-hypothesis. Rather, the relevance is diagnostic. Proprioceptive states are often taken to ground ownership directly because they enjoy immunity to errors through misidentification, yet even these states—whether construed as representational, non-objectifying perceptual, or sui generis bodily awareness—do not by themselves generate a phenomenology of ownership. This shows that ownership cannot be read off the intentional character of bodily awareness and therefore cannot plausibly be treated as a primitive phenomenal feature.

Moreover, multimodal integration reinforces the representational view. In actions like reaching and grasping, the brain matches proprioceptive data with visual and tactile information. When proprioceptive input is compromised, inaccuracies in reaching occur even if vision is intact. Conversely, manipulating visual input while preserving proprioception leads to predictable misreaching patterns. Neurons in parietal and premotor areas integrate these modalities, encoding spatial relationships like “object near the hand.” Conflicting cues lead to cross-modal recalibration and illusions, like the rubber-hand phenomenon. All of this suggests a unified egocentric spatial framework, where contents specify both body configuration and object location. It’s more compelling to see this as a representational system rather than mere sensorimotor patterns.

Proprioception also helps draw a clear line between mindless mechanisms and minded creatures. In many animals, proprioceptive signals run in closed-loop control systems that compare intended body positions with sensed ones and correct errors. Success and failure in posture, walking, and manipulation track whether proprioceptive states match real bodily states. This stable link between success, failure, and illusion is what we expect if proprioception uses internal states with correctness conditions tied to how the body and environment are. Purely phenomenological-enactive talk about sensorimotor coupling cannot make this distinction. Simple reflexes and complex proprioceptive systems both count as embodied interaction, but only the latter show the structured accuracy and error that support ascribing mind. The alternative view I defend takes this as central. Proprioceptive states represent the body as an object with parts, limits, and spatial relations. These states can be right or wrong, and their link with vision and touch grounds egocentric spatial layout and perceptual constancies.

Nothing in the present argument requires denying that proprioception involves a form of non-objectifying bodily intentionality, of the kind discussed by Husserl in terms of tendency, protention, and bodily orientation. It is therefore compatible with the view that proprioception constitutes a sui generis perceptual modality rather than a mere sensation or reflex. However, granting this point does not support the minimal self-thesis. Even if bodily awareness is perceptual in this non-objectifying sense, it does not follow that it includes a phenomenally manifest structure of mineness or ownership.

Proprioceptive illusions, body-schema research, multimodal integration, and feedback-based motor control all align more strongly with the representational view than Gallagher’s. While proprioception doesn’t represent distal objects, it provides objective, accuracy-based representations of the body and anchors our perception of the world. Next, we explore the concept of For-Me-Ness as ubiquity.

### For-me-ness as ubiquity

A key element of Zahavi’s defense of pre-reflective for-me-ness is the phenomenology of absorbed, world-directed activity. In this section, I do not treat absorption as a straightforward counterexample to the for-me-ness thesis. Rather, I use it as a methodological test case. The issue is not whether absorbed experience lacks mineness, but whether the for-me-ness thesis places any substantive empirical or phenomenological constraints on how such experience must appear.

In contrast, reflective self-awareness involves turning attention inward and explicitly recognizing oneself as the subject, such as saying, “I am reading” or “I am anxious.” Zahavi argues that reflection doesn’t create self-awareness from scratch; it simply makes explicit an inherent sense of mineness. Without this implicit mineness, absorbed experiences would be anonymous, which Zahavi finds absurd. Thus, reflection merely unveils what was already present, rather than generating it anew.

There are two distinct steps to consider. First, it’s true that during absorption, you don’t form any explicit, concept-based thoughts about yourself. Second, Zahavi concludes that the experience still includes a fundamental, primitive for-me-ness inherent to every conscious state. This second inference is what I want to challenge here. The data—how absorption feels and how we later describe it—don’t compel us to accept a consciously present sense of mineness (for-me-ness).

Zahavi faces first is the “refrigerator objection.” The objection says that claiming experience is always marked by pre-reflective self-consciousness is like thinking the light in a refrigerator is always on just because it is on whenever we open the door. The light is only potentially on, and opening the door, like reflecting, makes it actual. The analogy charges Zahavi with confusing the mind’s standing capacity for self-awareness with its constant, actual use. Zahavi’s reply is disappointing, because it assumes the point at issue, the subject-object model. He says the objection “might be tempting, but it is ultimately misleading since it remains stuck in the subject–object model” (2005, p. 63).

Nonetheless, Zahavi’s response is methodologically revealing. By rejecting the objection on the grounds that it presupposes a subject–object model, he effectively insulates the for-me-ness thesis from phenomenological challenge. Reports of diminished self-awareness during absorption can always be reinterpreted as evidence for a tacit, non-thematic form of mineness. As a result, no possible first-person description of absorbed experience—whether it emphasizes self-loss, anonymity, or complete world-directedness—can count against the thesis. This is precisely the structure of an irrevisable position.^5^

Experiential and phenomenological reports of absorption do not clearly support a primitive, consciously present for-me-ness. On the experiential side, research on flow shows that subjects describe deep absorption as a loss of self. Musicians, athletes, and climbers say “it was as if I wasn’t there anymore, only the playing remained,” or that “everything else disappeared, even the sense of ‘me’—there was just the music” (Csikszentmihalyi 1990, 53–54; Nakamura and Csikszentmihalyi 2002, 90). Studies of mind-wandering during reading report the same theme. Participants say their eyes kept moving across the page, but “I wasn’t really there,” noticing only later that they had turned several pages without awareness (Schooler et al. 2011).

Reports of automatic driving and fluent conversation show the same pattern. People say the car seemed to drive itself or that the words simply came out, and they highlight the absence of any felt self. Taken at face value, these spontaneous first-person reports describe immersion in the world and the fading of any sense of self. They do not indicate a primitive sense of mineness. That element enters only when we reinterpret the reports through a phenomenological framework that already assumes it. The fact that subjects later report a lack of self-awareness does not, by itself, entail the absence of phenomenal consciousness; the present argument concerns whether there is evidence for a phenomenally manifest structure of mineness, not whether experience occurred.

It is important to emphasize that the present argument does not rely on a strong Dreyfusian view on which skilled or habitual action is depsychologized or reduced to reflex-like processes. Skilled action may involve rich forms of control, sensitivity, and even conscious awareness, including peripheral or background forms of self-awareness. Nor does the argument infer the absence of phenomenal self-awareness from subjects’ later reports of being unaware of themselves, given the well-known distinction between phenomenal consciousness and access consciousness. The role of absorbed and skilled activity here is methodological: such cases show that the phenomenology of engagement can be described in multiple, equally compatible ways, none of which independently require positing a phenomenally manifest structure of mineness.

Cognitive science explains absorbed activity without invoking ongoing self‑awareness. Such episodes rely on fast, largely automatic world‑directed processing grounded in non‑conscious egocentric codes—head‑, eye‑, and body‑centered coordinates specifying object location and action guidance (Andersen et al. 1997, p. 615: “egocentric frames of reference for visuomotor transformation”). Skilled reading, driving, and playing instruments draw on entrenched sensorimotor routines, predictive control, and pre‑conscious body‑schemas rather than awareness of oneself as subject (Gallagher 2005, p. 141: “the body schema operates automatically and pre‑consciously”). Thus, during absorption, the subject is conscious only of the task and world; coherence arises from world‑directed content plus non‑conscious egocentric registration and motor control.

The sense that self‑awareness “must have been there” is retrospective. Reports like “I was completely absorbed” or “I drove home on autopilot” deploy self‑concepts to make post‑hoc self‑ascriptions grounded in sensorimotor traces and a stable self‑model. The episode is identified as theirs because the actions involved their body (Gallagher 2005, p. 136: “ownership is normally implicit in embodied action”). Nothing in these reports requires that a consciously experienced “quality of mineness” was present at the time.

In absorbed action, performance is highly efficient, error rates are low, and subjective effort is minimal, while explicit self‑related thinking is reduced or absent. Skilled agents in flow states—playing well‑practiced music, typing, or reading fluently—display smooth sensorimotor coordination and rapid online adjustments to environmental changes. Reaction times are fast, corrections occur without deliberation, and dual‑task interference remains low when the secondary task draws on different resources. Subjectively, people report being “lost in the activity,” “just doing it,” or that the task “does itself,” indicating that self‑related thought is unnecessary for precise performance.

However, just as Zahavi cannot independently establish the tacit presence of pre-reflective self-consciousness in all conscious activity, I cannot refute the for-me-ness thesis by appeal to phenomenological reports alone. This limitation is not a contingent weakness of the present argument, but a direct consequence of the structure of the for-me-ness thesis itself. Because the thesis is formulated so as to accommodate any possible phenomenology of absorption, its evidential status is not explanatory but hermeneutic.

Still, there is indirect neural evidence that bears against Zahavi’s position. Absorbed performance reliably engages sensorimotor, parietal, and cerebellar circuits that support egocentric spatial coding, action selection, and predictive control. Posterior parietal areas encode head-, eye-, and body-centered coordinates that guide movement and attention (Andersen et al. 1997, pp. 169–172). Premotor and cerebellar systems implement forward models and rapid error correction in skilled action (Miall et al. 1993, pp. 1185–1186; Wolpert and Ghahramani 2000, pp. 1214–1216).

By contrast, during deep engagement or flow states, neuroimaging studies consistently show reduced activity in medial prefrontal cortex and posterior cingulate cortex—core components of the default mode network associated with self-related and evaluative processing (Gusnard and Raichle 2001, pp. 701–703; Raichle et al. 2001, pp. 676–677; Ulrich et al. 2014, pp. 170–172). This pattern points to a system that relies on non-conscious egocentric registration and predictive control to guide action, while neural resources associated with explicit, reflective self-focus are attenuated.

Absorption thus illustrates, in a particularly clear way, why the debate over for-me-ness cannot be settled by phenomenological description alone and must instead be assessed by criteria such as explanatory constraint and theoretical economy.

### For-me-ness as pain

Pain is a central example for the sense of mineness. Zahavi claims that pain shows that consciousness is never anonymous. Pain is not a neutral change in how the world appears. It is always felt in the first person. As he writes, “I am not thematically aware of myself, yet there is still a minimal sense in which the experiences involved are experienced as my experiences” (Zahavi 2005, p. 15). In lived experience, pain is not an object you inspect. When you suffer, you do not step back and treat it as something you own. It shapes your whole way of being. Following Sartre, Zahavi notes that pain first appears through the disruption of the world. Sartre writes that one first lives “a world-in-pain,” not an inner object (Sartre, 1943, p. 114; see also Zahavi 2005, p. 16). Only with reflection do you turn it into “this pain of mine,” located “in my head” or “in my leg.”

This structure shows that the mineness of pain is pre-representational. The experience is not mine because I apply a self-concept or judge that “this state belongs to me.” There is no neutral sensory core that is subsequently tagged with an “I.” Rather, pain is lived from the outset as for me, without the mediation of any representational act. Husserl writes that to be a subject is “to exist in a mode of implicit self-awareness” (Husserl, 1973, p. 151), and that self-consciousness “manifests itself in its very flow,” without requiring a second act (Husserl, 1966, p. 83). Zahavi adopts this view under the label of pre-reflective self-consciousness: an intrinsic self-appearance of experience that requires no further representation (Zahavi 1999, pp. 15–18; see also Sartre, 1943, p. 114).

Mineness is not added to an otherwise anonymous experience. It is built into how the experience appears. The first-person character is not a separate feature, but part of what the experience is. Any episode of pain already involves this implicit “for-me-ness,” even if the subject never thinks “I am in pain.” This is why Zahavi rejects higher-order and conceptual views of self-awareness/self-consciousness. He argues that they place self-awareness too high, treating it as a later cognitive product rather than something given in experience from the start (Zahavi 2005, pp. 13–21).

On this view, pain is paradigmatically subjective. “Subjective” here does not mean private or metaphysically puzzling in the qualia sense. It means that the being of the pain cannot be separated from its being for a subject. Its identity depends on its first-personal givenness. In phenomenological terms, its mode of existence is that of a self-manifesting episode, not an object that could exist without appearing. To say what this pain is, one must say that it is lived as someone’s pain, experienced pre-reflectively as mine. Pain therefore offers a concrete case showing that subjectivity is a basic dimension of experience, in line with the phenomenological claim that “to be a subject is to be in the mode of being aware of oneself” (Husserl, 1973, p. 151; Zahavi 2014, pp. 25–29).

The key points are these. First, pain is not directed at the world in a way that leaves the subject out. Second, its mineness is not added later through a self-representation. Third, pain is usually treated as a paradigmatic subjective state. On closer inspection, though, pain is a representation that occurs in a subject, and its content targets a bodily condition, not the self. A pain episode represents that a specific bodily region is in a harmful or disturbed state, for example burning, stabbing, or throbbing, with a clear location and intensity. This content has correctness conditions. The experience is nonaccidentally accurate when the bodily condition that causes the token pain also serves as the truthmaker of its content, which matches your token-reflexive, truthmaker-dependent framework. The truth or falsity of the pain-experience depends on how the body actually is, not on any built-in “me” or “for-me-ness.” The subject is simply the point at which this bodily-directed, objectively assessable representation occurs.

However, you may wonder where the evidence is that pain can be captured in intentional terms. If pain were a subjective first-person for-me-ness with no objectification, there would be no clear sense in which it could be mistaken about anything. Nonetheless pain experience can be wrong about the state or location of the body, which shows that each pain episode carries content about how the body is.

Phantom limb pain is the clearest case. After amputation, many people feel strong pain “in” the missing hand or foot. From the first-person view, the hand feels as if it is burning or crushed, but there is no hand. The pain represents a damaged body part that does not exist. The natural reading is that the pain misrepresents the body. Referred pain shows the same point. During a heart attack, a person may feel sharp pain in the left arm or jaw and protect that area, even though the problem is in the heart. The pain attributes a damaging state to the arm that is not there. It is right that something is wrong, but wrong about where. This is a representational mistake.

Neuropathic and central pains add more support. In some cases, subjects feel burning or stabbing pain in a limb that is medically normal. The experience is “my leg is on fire,” but there is no tissue damage. The pain attributes a property the limb does not have. This is misrepresentation, not a neutral feel.

There are opposite cases too. In acute stress, people can suffer serious injury but feel little or no pain. The body instantiates the damage type that pain normally tracks, but the system fails to register it. This is also an error. Across these cases, pain is not only a feel in the subject. It presents itself as about the body, and it can be right or wrong. Having accuracy conditions of this kind is what makes a state representational.

A second set of considerations comes from how pain is used by the mind and how it is modulated by attention, belief, and context. Pure sensations would simply occur. Pain functions instead as an informational state. Sharp pain in your ankle leads you to alter your gait, protect the area, check it visually, and avoid loading it. You treat the pain as indicating that this part is in a harmful condition. When that representation is false, as in neuropathic pain, your behavior becomes systematically inappropriate.

Attention and expectation also shape pain in ways typical of representational states. Focusing on or away from a painful area changes the felt intensity. Expecting strong pain increases it, and expecting relief decreases it, as shown in placebo and nocebo effects. The mind does not receive a raw nociceptive signal. It combines sensory input with prior expectations and current focus, forming a representation of harm that ranges from mild to severe. Belief and interpretation show the same top–down influence. If a doctor tells you that a stabbing pain is harmless nerve irritation, the intensity can drop even when peripheral input stays the same. If you believe a mild sensation signals something serious, the experience can intensify. Pain here behaves as a contentful state whose representational content is shaped by higher-level factors.

The distinctive sense in which pain is often described as paradigmatically “mine” can be explained without positing a primitive phenomenal structure of ownership. Pain is not merely a representation of bodily disturbance; it is embedded in a defensive–homeostatic system that assigns high motivational and affective priority to certain bodily states. Nociceptive signals are coupled with threat valuation, urgency, and action readiness, recruiting protective behaviors, attentional capture, and avoidance responses. Through re-entrant interactions among sensory maps, interoceptive monitoring, and motivational systems, pain comes to dominate the organism’s practical field.

On this view, what phenomenologists describe as the mineness of pain reflects its role in regulating survival and bodily integrity, not the presence of an additional phenomenal ingredient. Pain feels inescapably personal because it is tightly anchored in systems that track bodily threat and guide immediate action, not because it carries an intrinsic, pre-reflective mark of ownership. Mineness, in this case, is an effect of how pain is functionally integrated into the defensive and homeostatic architecture, rather than a basic feature of conscious experience as such.

This functional embedding of pain within motivational and defensive systems also helps explain why pain is highly sensitive to interpretation and context. Therapeutic work in chronic pain supports this pattern. When subjects change how they understand the pain—for example, from “this flare means my back is being damaged” to “this is uncomfortable but not harmful”—pain can decrease sharply and stay lower over time. The causal pathway runs through meaning. Changing what the pain is taken to signify changes the experience itself. This is what we expect if pain has representational content about bodily conditions.

The interpretation of depersonalization and derealization proposed here relies on the same architectural framework developed in Sect. 7. On this view, experiences can remain conscious and world-directed while failing to be integrated into self-modelling and affective systems in a manner that yields a stable sense of ownership.

### For-me-ness in DPD

Several recent accounts explain Depersonalization–Derealization Disorder (DPD) by appealing to purportedly global structural features of consciousness, such as mineness, present or actual character, or an all-pervasive affective flow (Billon 2016; Gerrans 2017; Ciaunica et al. 2021). On these views, a healthy subject’s conscious states are typically marked by a sense of being mine, by being experienced as occurring now in the actual world, or by being permeated by an affective tone that tracks personal relevance. DPD is then construed as a disorder in which one or more of these global structural features are disrupted.

Billon associates distinct clusters of DPD symptoms with alterations in mineness, present character, and actual character. Gerrans interprets the disorder as a failure of the global affective flow that ordinarily confers both mineness and significance. Ciaunica and colleagues appeal to a disturbance of pre-reflective self-consciousness and a breakdown of experiential transparency (see also Knappik 2022, pp. 413–415). Despite their differences, these approaches share a common explanatory strategy: they account for the alienation characteristic of DPD by positing a deep and singular disruption in the basic structure of conscious experience itself.

Zahavi and Gallagher offer a phenomenological reading of DPD. They treat it as a limit case that makes explicit what is usually tacit in conscious life: a basic sense of self-presence and a familiar, meaningful world. Subjects often report that their thoughts, emotions, or movements do not feel like theirs, or that they observe themselves from the outside. Zahavi notes that people describe their experience as “strangely alien, detached, or as if they were happening to someone else,” however he argues that such reports “are intelligible only if we accept that under normal circumstances experiences manifest a pre-reflective for-me-ness” (Zahavi 2005, p. 134).

Gallagher interprets depersonalization as a disturbance of the lived body and agency, a breakdown in the pre-reflective sense of being an embodied subject who moves, feels, and perceives (Gallagher 2005, 2007). Both claim that even the sense that “this is not really me” remains given from a first-person point of view, so it presupposes minimal selfhood rather than denying it.

In line with this view, Zahavi and Gallagher reject representational accounts of DPD. Zahavi argues that the sense of detachment shows that for-me-ness may vary in strength but cannot be missing, since it is not a representational layer added to otherwise anonymous states (Zahavi 2005). Gallagher makes the embodied version of the same point. He holds that depersonalization is not a belief but a disruption in the integration of proprioception, affect, and agency. The subject no longer feels fully present in her movements and actions (Gallagher 2007). What fails is not a self-model but the implicit, sensorimotor, and affective grip through which the self is normally lived.

A similar account applies to derealization. People say the world feels flat, distant, empty, or unreal, as if seen through a barrier or as if they were walking through a stage set. Zahavi notes that the world does not vanish. It stays present, but it shows up with reduced affective and practical force (Zahavi 2005). Gallagher, using an enactive version of the neo-phenomenological view, says that derealization comes from a break in embodied sensorimotor coupling. Objects lose their normal salience, affordances, and pull for action (Gallagher 2005). In both views, the experience still belongs to a first-person stance. If the world feels unreal, it is still unreal for someone.

Some theorists claim that clinical data from DPD do not support the idea that every conscious state has built-in for-me-ness. Many depersonalization reports show vivid, structured, and emotionally charged experiences that lack any felt sense of ownership. Sierra and Berrios describe a patient who says, “I watch my hands move, but it doesn’t feel like I’m doing it” (Sierra and Berrios 1998, p. 379). Non-egological and higher-order thought theorists do not see this as a disturbance inside a self-manifesting experience. They say these reports show intact first-order motor and perceptual states that lack the higher-order monitoring that normally links them to the subject. On this view, mineness is not basic. It is a representational add-on that can fail. Rosenthal argues that when higher-order states do not arise, the first-order experience stays conscious but no longer feels like it belongs to the subject (Rosenthal, 1997, pp. 735–736). Carruthers also holds that the sense of ownership can “drop out of phenomenology” while consciousness remains (Carruthers 1996, pp. 152–158). For these theorists, depersonalization is direct evidence that consciousness can continue without for-me-ness.

A similar tension shows up in trauma-related depersonalization. Simeon and colleagues describe a patient who says, “I feel my legs moving, but they don’t feel like mine” (Simeon et al. 2003, p. 792). Zahavi sees this as a disturbance within an otherwise active dimension of ipseity (Zahavi 2005, pp. 165–169). Representational theorists disagree. They argue that ownership is not built into experience. Metzinger claims that alienation experiences reveal a failure in the phenomenal self-model that produces bodily ownership, while low-level perceptual and sensorimotor processes continue as normal (Metzinger 2003, pp. 443–445). On this view, depersonalization shows that mineness is a constructed and defeasible layer that can disappear while consciousness stays intact.

Assume, for the sake of argument, that intuitions supporting or rejecting phenomenological for-me-ness end in a stalemate. The next step is to look at the broader empirical picture. DPD is a chronic condition with a wide and variable symptom profile (Knappik 2022, pp. 411–413). Core complaints include emotional numbing, reduced pleasure and pain, mechanical or automatic actions, alienated thoughts like ‘my thoughts have a life of their own,’ altered episodic memory, flat imagery, feelings of unreality or alienation from oneself, bodily disownership, out-of-body experiences, distortions of body size, derealization, spatial and auditory distortions, fragmented time, déjà-vu, jamais-vu, and cognitive and attentional impairments (Knappik 2022, pp. 411–414).

Knappik argues that mineness-based models impose theoretical commitments that the data do not require (Knappik 2022, pp. 415–416). The claim that all conscious states necessarily carry a mark of pre-reflective mineness is contested, and even those who accept pre-reflective self-consciousness, such as Zahavi (2025), deny that it can fail in DPD. This creates a tension: if mineness cannot fail, then DPD cannot alter it; but if DPD does alter it, then the universality of mineness is undermined.

Beyond these conceptual worries, global for-me-ness models do not explain how a single structural disturbance could produce the many specific anomalies found in DPD, including spatial distortions, observer-memory, imagery deficits, thought alienation, disembodiment, and fragmented time (Knappik 2022, pp. 416–417). Gallagher reads depersonalization as a disturbance of embodied agency, but empirical work shows that agency draws on several cues, including motor prediction, sensory feedback, and context, and these cues can compensate for one another. Losing affective cues alone cannot eliminate agency (Moore and Fletcher 2012, pp. 60–63; Knappik 2022, p. 416). PTSD studies also show that emotional numbing can occur without producing the wider DPD pattern. This weakens Gerrans’s view that affective flow globally structures mineness (Frewen et al. 2015, pp. 6–7; Knappik 2022, p. 416).

Symptom variability reinforces this problem. If mineness or any other global feature were the sole point of failure, symptom profiles should be stable and unified. They are not. Some patients show marked depersonalization with minimal derealization; others show the reverse; many display only a few symptoms; and symptoms often split in ways incompatible with a unified loss of for-me-ness (Sierra et al. 2005; Simeon et al. 2008; Knappik 2022, pp. 412–413, p. 417). This variability undermines the idea that a single structural disturbance explains the syndrome.

A more empirically grounded view treats DPD as a disorder marked by several partly independent impairments (Knappik 2022, pp. 419–421). One patient may show strong emotional and memory disruptions without spatial distortions or derealization. Another may show spatial, bodily, or temporal disturbances while remaining affectively intact. This pattern fits the observed heterogeneity. Under stress or trauma, different defensive or regulatory systems may fail in different combinations, weakening local self-relations, including bodily, affective, spatial, memory, and temporal ones. This does not require a global collapse of consciousness. When enough of these local relations deteriorate, the subject may report global-sounding feelings of unreality and self-loss. These reports arise from the combined impact of specific disruptions rather than from the loss of a single global feature such as mineness. On this interpretation, the characteristic loss of experiential vitality in depersonalization reflects a disruption of affective and motivational integration rather than a loss of world-directed content, a pattern that affect-centered frameworks such as MEM are well suited to capture. As Adam Bradley puts it:

Subjects with DDD have affective experiences, but of a strange sort. Their affective experiences no longer directly motivate them to act and think in typical ways. As a result, subjects feel alienated from their own actions and thoughts. Thus, this view posits that depersonalized subjects are still able to experience affective states such as a feeling of joy in seeing a loved one. However, instead of immediately and unreflectively prompting appropriate thoughts and behaviors, this joy experience leaves the subject feeling cold and unmoved—not immediately motivated to perform the sorts of actions typically characteristic of feeling joy. As a result, the subject feels detached from their own affective experience, even to the point of claiming that they cannot experience joy at all (2026, p. 161).

On this more local and empirically grounded view, you do not need to assume that all normal consciousness carries a global mark of mineness or that DPD reflects the failure of such a structure. The syndrome’s phenomenology and variability fit better with fine-grained disruptions in the processes that place the subject in space, time, causality, embodiment, affect, and value. Global mineness (for-me-ness) models add theoretical cost without adding explanatory gain beyond what the empirical pattern of DPD already shows. This raises a further question about whether mineness appears early in development and how it functions in conditions such as autism.

### For-me-ness in infancy and autism

A further strand of the phenomenological case for pre-reflective for-me-ness draws on developmental and clinical research on infancy and autism. Gallagher first introduced this line of argument, and Zahavi later incorporated it into his own view. The central claim is that very young infants possess a primitive, pre-reflective bodily self-awareness, and that atypical features in autism reflect disruptions of this basic structure. On this interpretation, the empirical data are taken to show that a minimal experiential self, or ipseity, appears before conceptual, linguistic, or social abilities emerge.

Gallagher argues that every experience carries a pre-reflective sense of ownership, and that early infancy already shows an embodied form of mineness (Gallagher 2000, p. 15). He cites neonatal imitation, early body awareness, and contingency detection as evidence that a basic awareness of the body as the site of experience and action comes before self-concepts and social cognition (Gallagher 2005, pp. 173–176). Zahavi uses these developmental results to support a distinction between a minimal experiential self, grounded in pre-reflective bodily self-awareness, and richer narrative and socially extended forms of selfhood (Zahavi 2005, pp. 129–131). On this view, both infancy and autism suggest that a primitive for-me-ness is present from birth and remains even when later social capacities diverge.

A closer look at the empirical literature supports a weaker conclusion. Developmental and clinical findings reveal early, sophisticated forms of egocentric, body-centered processing, but they do not show that infants or autistic individuals experience their states as mine or possess a phenomenological for-me-ness. When we describe the empirical phenomena without adding phenomenological assumptions, a wider range of interpretations becomes available. These include accounts grounded in sensorimotor contingency detection, multisensory integration, action–perception coupling, and predictive processing. None of these mechanisms require an intrinsic phenomenal structure of mineness.

Infant research illustrates this point. From birth, infants track temporal and spatial contingencies between movements and sensory feedback. Classic studies show that newborns differentiate synchronous from asynchronous proprioceptive–visual stimulation and prefer contingent feedback tied to their actions (Bahrick and Watson 1985, p. 205). Infants also notice when correlations between limb motion and visual feedback break down and respond with surprise or disorganized searching (Rochat and Morgan 1995, p. 147). By two months, they adjust sucking and reaching in line with real-time proprioceptive cues (Rochat 2003, pp. 717–720). These findings show early integration of proprioception, touch, and vision within an egocentric frame. They are compatible with non-conscious sensorimotor registration and predictive updating. Calling these achievements for-me-ness adds a phenomenological layer the data do not require.

More recent work confirms this pattern. Studies using body-swap and full-body illusions show that infants have partial, noisy, and easily altered body-representations shaped by self–other contingency manipulations (Filippetti et al. 2013, p. 110). Infants respond differently to visuotactile synchrony applied to a virtual body versus their own, suggesting that body-ownership signals arise from multisensory integration rather than from a primitive felt mineness. Predictive-coding models can explain these effects through learned mappings between actions and sensory outcomes. No phenomenological assumption is needed.

Autism research shows the same structure. Some phenomenologists argue that autism supports the distinction between minimal and narrative selfhood, since autistic individuals retain a basic experiential self despite social-cognitive differences. But empirical findings point instead to specific differences in multisensory integration, predictive inference, interoceptive precision, and social cue processing. These differences do not concern the presence or absence of a primitive for-me-ness.

Autistic individuals often show reduced integration of visual and proprioceptive cues (Haswell et al. 2009, p. 421), altered weighting of interoceptive signals (Garfinkel et al. 2016, p. 412), and differences in synchrony detection across modalities (Foss-Feig et al. 2010, p. 1009). They may also show atypical responses in rubber-hand-illusion tasks, sometimes displaying heightened susceptibility and sometimes resistance depending on stimulus parameters (Paton et al. 2012, p. 302). Predictive-processing models explain these effects through variations in precision-weighting and hierarchical inference. Nothing here suggests that autism involves alterations in a fundamental experiential ipseity.

Social differences in autism also admit non-phenomenological explanations. Distinct profiles in gaze processing, facial-expression recognition, and emotional prosody correlate with differences in attention, learning, and sensory integration, not with changes in a primitive experiential self (Chevallier et al. 2012, p. 4). Differences in agency judgments can be explained by atypical predictive modeling of action consequences (Zalla et al. 2015, p. 27). Autistic individuals can develop different patterns of bodily ownership, agency, and social interaction, but the most empirically grounded explanations appeal to perceptual, sensorimotor, and inferential mechanisms rather than to variations in for-me-ness.

Together, the infant and autism data support a three-part view. First, infants show early, non-conscious, body-centered registration of proprioceptive, tactile, and visual events shaped by sensorimotor contingencies and predictive learning. Second, self-models grow more complex through social interaction, language, and cognitive development, which stabilizes richer forms of self-representation.

Third, autistic individuals show specific differences in multisensory integration, interoceptive weighting, and predictive inference that shape body-awareness and social cognition. None of these findings require a phenomenologically robust pre-reflective for-me-ness in infancy or autism. Applying the notion of ipseity to these data projects a philosophical idea onto the empirical record rather than deriving it from the evidence. This brings us to how far non-conscious egocentric processes can go on their own.

### Egocentric registration as weak for-me-ness

Neo-phenomenological defenses of for-me-ness typically treat mineness as a primitive feature of experience. On such views, each conscious state includes, over and above its worldly content, a pre-reflective sense of being mine. I propose an alternative that preserves what is explanatorily valuable in this tradition—perspectival organization, bodily anchoring, first-person authority, and sensitivity to clinical data—while rejecting the postulation of a sui generis phenomenal ingredient of mineness. The basic claim is that once we take seriously (i) that perceptual and bodily states have world-involving content, and (ii) that organisms non-consciously encode and integrate these states within an egocentric architecture, no further phenomenal posit is required.

The argument begins at the level of subpersonal organization. A large empirical literature describes perception and action in terms of egocentric, body-centered, and action-centered coding. Posterior parietal regions integrate visual, proprioceptive, tactile, and vestibular signals into head-, eye-, limb-, and body-centered maps specifying the spatial relations between objects and the organism (Andersen et al. 1997). These coordinate frames are flexible and task-sensitive, as shown by work on reversible cortical inactivation and intracranial recordings (Batista et al. 1999; Chang and Snyder 2010). Such representations are perspectival in an information-processing sense, yet there is no evidence that they carry any phenomenal marker of ownership or mineness.

Motor and cerebellar systems further implement forward models that predict the sensory consequences of action and enable rapid error correction (Miall et al. 1993; Wolpert and Ghahramani 2000). Distinctions between predicted and unpredicted sensory input underwrite familiar agency phenomena, such as sensory attenuation for self-generated movement (Blakemore et al. 1998). These mechanisms are egocentric and action-specific, but again there is no indication that they generate a felt sense of ownership.

Interoceptive and nociceptive signals are likewise mapped into body-centered representations in insular and cingulate cortices, supporting affective and homeostatic regulation (Craig 2009). Lesion studies show that such interoceptive representations can be selectively disrupted without reports of any loss of subjective ownership (Karnath et al. 2005; Salvato et al. 2020). These findings reinforce the point that egocentric representational structure does not require a primitive phenomenal marker of mineness.

Together, these systems realize what Gallagher has called the body schema: a dynamic, non-conscious representation of posture, boundaries, and capacities that guides action automatically (Gallagher 2005). Contemporary work shows that body-schematic processing underlies tool use (Maravita and Iriki 2004), spatial attention (Konen and Kastner 2008), and online locomotor control (Matthis et al. 2018). This architecture is thoroughly egocentric, yet it does not appear in experience as an additional phenomenal dimension of ownership.

On this basis, agents develop more enduring self-models: representations of their bodies, traits, memories, dispositions, and social identities. These models are shaped through learning and interaction, and they support autobiographical memory, planning, and first-person thought. Work on self-referential processing shows that judgments about one’s own traits robustly activate medial prefrontal cortex (MPFC) and yield enhanced memory performance (Kelley et al. 2002). Later studies show that this network integrates self-related information across time (D’Argembeau et al. 2014), tracks personal traits and goals (Wagner et al. 2012), and updates the self-model in light of feedback (Molnar-Szakacs and Uddin 2013). These models let the organism treat perceptual and bodily states as components of its own cognitive system. As Burge argues, entitlement to perceptual belief depends on reliable world-tracking, not on phenomenally accessible ownership markers (Burge 2003). Much of what neo-phenomenologists want from for-me-ness is thus supplied by (a) world-involving representational content and (b) integration into egocentric and autobiographical models.

A further layer is the Bayesian predictive self-model. Predictive-processing theories hold that the brain minimizes prediction error by generating probabilistic hypotheses about the world and about itself (Friston 2003, 2009, 2017; Hohwy 2013). Perception is active prediction, and mismatches with input drive model revision. This architecture extends to interoception and proprioception and generates a dynamic model of the organism’s bodily states and likely actions (Seth 2013; Allen and Friston 2018). Friston describes this as “self-evidencing,” where the system continually tests hypotheses about its viability and future states. Seth interprets this as a model that produces the sense of being a self (Seth 2021). More recent work shows that interoceptive prediction errors shape affective self-modelling and the experience of presence and ownership (Barrett and Simmons 2015; Smith et al. 2021).^6^

Because predictive systems require internal coherence, self-related signals must be integrated rather than treated as isolated tags. Fragmented cues would generate persistent prediction error. The architecture therefore pressures the system to bind transient episodic states (“I am in pain,” “I intend to move,” “I recall”) into a more stable, temporally extended self-model. Work on “superordinate schemas” supports this: MPFC organizes self-relevant information into a unified structure that stabilizes self-reference and improves memory (Northoff & Bermpohl, 2004). On my interpretation, this schema is the higher-level predictive self-model.

Egocentric registration by itself does not amount to a phenomenology of ownership. Perceptual and bodily states can guide behavior while remaining unexperienced as mine. Ownership emerges only when egocentrically encoded content is integrated into a self-model that supports memory, report, deliberation, and action monitoring. This integration is contingent and context-sensitive. When it occurs, experiences become accessible as part of the subject’s own history. When it does not, conscious content may remain world-directed and action-guiding without a sense of ownership.

Although the alternative developed in this paper makes frequent use of ideas drawn from predictive processing—such as predictive self-modelling, self-evidencing, and interoceptive prediction error—the argument does not depend on the truth of predictive processing as a general theory of the mind. Predictive processing is invoked here as one possible realization of a more general egocentric architecture, not as its theoretical foundation. What matters for the present argument is the existence of mechanisms that integrate perceptual, bodily, and affective information in a manner centered on the organism and capable of supporting action, regulation, and learning, without positing a primitive phenomenal mineness.

Other frameworks can equally serve this role. In particular, more biologically and affectively grounded approaches, such as the Motivated Emotional Mind (MEM) model, offer an alternative way of characterizing how interoceptive signals, re-entrant sensory mappings, and motivational–affective valuation jointly structure experience. Such models may be especially well suited to explaining phenomena like pain and depersonalization, where affective significance and bodily regulation play a central role, and they do so without appealing to an intrinsic, pre-reflective sense of ownership. In this respect, affect-centered and embodied models such as MEM can be understood as implementational accounts of how egocentric sensory maps, interoceptive monitoring, and motivational valuation jointly constitute what phenomenologists describe as subjective appearance, rather than merely causing it.

The deflationary argument advanced here is therefore not tied to predictive processing in particular. It targets the assumption that the explanatory work in these domains requires positing a built-in phenomenal for-me-ness, an assumption that both predictive and affect-centered frameworks can dispense with.

Egocentric registration by itself does not amount to a phenomenology of ownership. Perceptual and bodily states can be encoded in body-, limb-, or action-centered frames and guide behavior without any experience of those states as mine. On the present view, ownership appears only when egocentrically encoded content becomes integrated into a self-model that is used for memory, report, deliberation, and action monitoring. This integration is not automatic or constant. It depends on whether information about the ongoing state is taken up by systems that track the subject as a persisting agent across time and contexts. When this uptake occurs, the experience can be accessed, recalled, reported, and evaluated as part of the subject’s own history, and a phenomenology of ownership may emerge. When it does not, conscious content can remain world-directed and action-guiding without being experienced as owned.

This account predicts that ownership is task-, affect-, and context-dependent rather than a standing feature of consciousness. In absorbed activity, skilled performance, and certain clinical conditions, egocentric content guides perception and action while remaining largely isolated from self-modelling resources, resulting in conscious experience without a marked sense of ownership. By contrast, in reflective contexts, emotionally salient situations, memory formation, or explicit self-assessment, the same kinds of contents are more likely to be integrated into the self-model and experienced as one’s own. Ownership, on this view, is not a primitive phenomenal ingredient but a contingent outcome of how egocentric content is functionally integrated within the cognitive architecture.

A final clarification concerns the familiar “hard problem” objection. The present account does not seek to reduce phenomenal character to physiological activity, nor to explain consciousness in purely physical terms. However, it rejects the assumption—most clearly articulated by Levine—that there is a principled explanatory gap between mechanisms and appearance. As Papineau argues, once the relevant empirical identifications are in place, there is no further fact left to be explained (Papineau, 2002). The demand for an additional explanation reflects a residual neo-dualism of properties rather than a genuine explanatory deficit.

This is not to deny that consciousness presents deep explanatory challenges. It is to deny that these challenges take the form posited by the hard problem. On the present view, egocentric registration, self-modelling, and affective–motivational integration constitute the functional organization in virtue of which experiences are first-personal. This is a constitutive claim within a naturalistic framework, not an eliminativist one.

Finally, this proposal differs from both higher-order and global workspace theories. Unlike higher-order views, ownership does not require a representation of oneself as being in a mental state. Unlike global workspace accounts, ownership is not conferred uniformly by global access. Conscious contents may be locally available for perception and action without being integrated into self-related processing. This explains how conscious experience without ownership is possible—an outcome obscured by views that treat mineness as either a necessary higher-order accompaniment or a ubiquitous consequence of global broadcasting. Here is the argument:

1. Any conscious experience is organized around a single point of perspective and belongs to a subject in a minimal sense; conscious experience is therefore *for someone*.
2. Empirical research in perception, action, and interoception shows that perceptual, bodily, and affective states are encoded non-consciously in egocentric, body-centered, and action-centered representational formats.
3. These egocentric representational formats are sufficient to explain perspectival organization, bodily anchoring, sensorimotor coordination, and the guidance of perception and action.
4. Egocentric representational formats operate without representing the subject as a persisting individual and without encoding a distinctive phenomenal feature of mineness.
5. Cognitive systems sometimes treat certain perceptual, bodily, and affective states as belonging to the subject in a practical sense: such states are stored in autobiographical memory, guide deliberation, regulate action, and enter first-person reasoning.
6. Treating states as belonging to the subject in this practical sense requires the functional integration of egocentrically encoded content into a temporally extended self-model (the EGO-file).
7. The functional integration described in P6 does not add a new phenomenal ingredient to experience but alters how egocentrically encoded content is used, retained, and coordinated across cognitive systems.
8. Variations in phenomena commonly described as “ownership” systematically track whether egocentrically encoded content is integrated into such a self-model across tasks, affective contexts, and clinical conditions.
9. Positing a primitive phenomenal property of for-me-ness over and above egocentric registration and self-model integration does not explain these variations and adds no independent explanatory value.

C- Therefore, conscious experience is subject-involving in virtue of egocentric registration alone. The strong phenomenal notion of for-me-ness should be rejected. What exists instead is a *weak sense of for-me-ness*, understood as ownership in Guillot’s sense, which consists in the functional integration of egocentrically encoded content into a self-model rather than in a sui generis phenomenal feature of experience.

### Objections and replies

*Objection-1*. A phenomenologically informed reply to my empirical objections might begin by rejecting the idea that subpersonal mechanisms can function as explanatory competitors to for-me-ness. They would argue that the point is not to deny these mechanisms or their importance, but to note that they operate at a different explanatory level from the one at which for-me-ness is located. When I appeal to egocentric registration, predictive self-modelling, hierarchical inference, or sensorimotor integration, the phenomenologist accepts the empirical claims as far as they go.

They maintain, however, that these mechanisms describe the conditions under which a conscious episode is produced, not how it appears to a subject. For them, for-me-ness is not an extra feature within experience but the formal structure under which any feature appears. Empirical models of registration or integration are not incorrect, but they remain silent about lived manifestation. On this reply, phenomenologists invert my argumentative posture. They claim that empirical descriptions cannot, by their nature, cover the structural dimension that phenomenology seeks to articulate.

*Reply-1*. A sustained reply to the phenomenologist’s claim that empirical models and phenomenological analyses occupy distinct explanatory levels begins by challenging the insulation thesis at its core. Predictive self-modelling, egocentric coding, multisensory integration, and bodily registration do describe enabling conditions, but this does not mean they say nothing about subjective appearing. On my view, subjective appearing is the way world-directed perceptual content is implemented within an organism’s egocentric and predictive architecture. To claim that these models reveal only causal production and never the subject-side of experience assumes what is under dispute: that subjective appearing must be something beyond the contentful state and its embedding in a self-specific system.

The phenomenologist’s strict divide between appearance and mechanism rests on a dualist assumption that my view rejects. If conscious appearing is nothing more than the tokening of world-involving content in a truthmaker-dependent, egocentrically indexed system, then empirical models do not sit outside appearance, they help make it what it is. The claim that there is a separate phenomenological level is not a discovery, it is a theoretical presupposition. And the very phenomena appealed to by phenomenologists are already accounted for within a simpler and more unified framework.

*Objection-2*. A phenomenologist might also say that my view treats for-me-ness as if it belonged to mechanisms or behavioral patterns. They hold that no amount of detail about vestibular cues, predictive priors, multimodal maps, or autobiographical modeling can explain the subject-side of experience. These models show how a system moves through its environment, updates its states, or identifies itself as the source of its actions and thoughts. They do not show how the world appears as experienced. For-me-ness is not another item in experience, it is the lived way anything appears. The phenomenologist will say that a deflationary architecture only gives you an ‘as-if’ shadow of subjectivity. Without the structural element they call for-me-ness, the subject shows up too late, inferred from processing patterns rather than present in the immediate appearance.

*Reply-2*. The phenomenologist’s objection assumes that experience has a subject-side beyond world-directed content embedded in an egocentric, truthmaker-dependent system. On my view, there is no extra phenomenal ingredient. The subject-side is just the organism tokening accurate, world-involving content within its predictive and egocentric architecture. Once that relation holds, the mode of appearance is fixed. The split between ‘lived appearance’ and the mechanisms that enable appearance is a theoretical stipulation, not something revealed in experience. If appearance just is the tokening of content inside the organism’s predictive, egocentric system, then empirical models do not create an ‘as-if’ subjectivity. They constitute it. The idea that the subject must ‘show up earlier’ presupposes a special phenomenal factor that no phenomenological report isolates.

Nothing in the objection shows that egocentrically indexed, non-accidentally accurate content fails to fix how the world is given. The phenomenologist’s claim of an irreducible self-present structure is unmotivated, and the deflationary account explains the relevant phenomena without positing any further layer.

*Objection-3*. The phenomenologist claims that while one can conceptually imagine consciousness without mineness, such imagining covertly imports the very standpoint it tries to exclude. When you picture a conscious state belonging to no one, you do so from a third-person view, which phenomenology denies is the stance from which lived experience is disclosed. Consciousness is always first-personal in its givenness, and removing that dimension leaves you with something that no longer counts as consciousness. Anonymous experience is not conceptually impossible, but it lacks phenomenological sense, because to grasp an experience as given you must already occupy a perspective from which it is encountered.

*Reply-3*. The phenomenologist’s claim about anonymous consciousness does not succeed. She claims that imagining experience without mineness secretly relies on a first-person stance, because imagining always involves a subject-pole. But this confuses the trivial fact that some organism hosts an experience with the stronger claim that the experience has a self-present phenomenal dimension. Anonymous consciousness does not require a view from nowhere. It holds that world-directed content can be conscious without also feeling like mine. Infants, non-linguistic animals, and people in depersonalization episodes show exactly this pattern: experience occurs, content is present, the world is given, yet there is no felt mineness. The phenomenologist insists that such cases must contain tacit self-givenness but offers no evidence. The charge of phenomenological incoherence simply restates the point at issue.

*Objection-4*. Objection 3 also shapes the phenomenologist’s reply to my discussion of depersonalization, derealization, infancy, and autism. She accepts that these cases involve severe disruptions of embodiment, affect, spatiality, or social attunement, but claims that such disruptions presuppose a structural self-presence rather than challenge it. Depersonalization is taken to reveal a disturbance of the normally tacit self-givenness of experience. When subjects say that their thoughts, emotions, or movements feel alien, the phenomenologist treats this not as evidence that mineness is absent, but as proof that mineness must still function as the background against which alienation makes sense.

*Reply-4*. The claim is that these conditions reflect distorted modes of an underlying ipseity rather than experiences without mineness. However, the empirical record shows multiple dissociable impairments—affective blunting, spatial distortion, anomalous agency, mnemonic fragmentation, interoceptive attenuation, sensory deregulation, and predictive instability. None of these require positing a structural self-present background. The heterogeneity of symptoms argues against a unified phenomenal layer. Phenomenologists infer a universal ipseity because they start from the assumption that mineness is necessary, then reinterpret all disruptions as partial degradations. A pluralistic, empirically grounded account explains these effects through local failures in egocentric integration, interoceptive precision, body-schema calibration, memory, affect, and higher-order self-ascription, making a separate experiential layer redundant.

*Objection-5*. The same reasoning applies to infancy and autism. Phenomenologists see early body-awareness, contingency-detection, and social interaction not as building selfhood from scratch, but as gradual expressions of a pre-existing structural ipseity. Empirical measures of contingency-tracking or multisensory integration capture developmental or clinical changes but cannot determine whether the phenomenological mineness they posit is actually present.

*Reply-5*. When the phenomenologist claims that predictive, representational, or sensorimotor models cannot bridge an ‘appearance gap,’ she presupposes the very point under debate: that phenomenal character includes an irreducible, self-present dimension. I argue there is no such gap. Subjective experience is fully captured by a state’s content being non-accidentally accurate relative to its truthmaker and instantiated within egocentric, predictive, and self-modelling structures. For-me-ness is assumed as intrinsic only because phenomenology conceives consciousness as self-manifesting, but this is a theoretical assumption, not an empirical datum. Predictive models leave out nothing except the phenomenologist’s own posit.

## Conclusion

This paper has critically examined the phenomenological claim that every conscious experience includes a primitive, pre-reflective “for‑me-ness.” I argued that the only evidential support for this thesis relies on the interpretive framework it is meant to justify. Once we separate (a) egocentric or perspectival structure, (b) ordinary self-ascription and autobiographical practices, and (c) the stronger claim that consciousness intrinsically includes phenomenal mineness, it becomes clear that the third element lacks independent empirical support. Putative independent evidence cited to reveal for‑me‑ness—absorption, pain, bodily awareness, depersonalization, derealization, infancy, and autism—are better explained by non-conscious egocentric registration, world-directed content, predictive self-modelling, and concept-involving self-ascription.

Absorption and flow involve intensified world-directed processing with reduced explicit self-focus, accounted for by egocentric mapping, predictive control, and retrospective self-interpretation. Pain, often treated as paradigmatic of mineness, shows location-specificity, misrepresentation (phantom limb, referred pain), and modulation by attention and belief—features consistent with representational content whose accuracy depends on the coincidence of cause and truthmaker. Depersonalization and derealization decompose into dissociable impairments of affect, interoception, spatial processing, agency, bodily representation, memory, and attention; their heterogeneity conflicts with the idea of a broken universal mineness. Infant and autism research reveals early multisensory integration, contingency detection, and predictive learning, but no independent evidence of pre-reflective phenomenal ownership. Across these domains, the empirical record supports egocentric and predictive mechanisms, not a sui generis experiential layer.

I then offered an alternative account that retains what is indispensable: perspectival structure, bodily anchoring, first-person authority, and stable self-models, while eliminating primitive for-me-ness. Conscious states are world-involving content instantiated within egocentric, predictive architectures. Head-, eye-, limb-, and body-centered frames integrate perceptual and interoceptive signals, generate forward models, and monitor deviations to produce non-accidentally accurate experiences. Higher-order and autobiographical processes stabilize self-models for reasoning, self-ascription, and narrative. On this view, the sense that experiences are mine is not a fundamental feature of phenomenal character but a downstream consequence of content processing and self-model accessibility. All explanatory work attributed to for‑me‑ness is discharged by this architecture; no additional experiential layer is required.

## Data Availability

No datasets were generated or analysed during the current study.

## Footnotes

1 Some authors distinguish between for-me-ness and what might be called for-someone-ness, understood as the mere fact that an experience has an experiencer or is available from a first-person point of view, without involving awareness of oneself as a self or as the owner of the experience (see, e.g., Guillot 2017). The account developed in this paper is compatible with this weaker notion. Egocentric registration and the EGO-file framework explain how conscious states can be organized around a subject and function as that subject’s experiences without positing a phenomenally manifest structure of mineness or ownership.

2 Recent enactivist approaches to cognition extend and radicalize this point. They model perception and action in head-, eye-, and body-centered coordinates and emphasize sensorimotor contingencies and affordances. Enactive and embodied approaches, often drawing on phenomenology, also hold that cognition unfolds in a situated, organism-relative space of possibilities rather than in an abstract, featureless arena. Francisco Varela, Evan Thompson, and Eleanor Rosch describe cognition as “enaction,” the bringing forth of a meaningful world through sensorimotor coupling (Varela et al. 1991, pp. 173–178). Alva Noë argues that “perception is not something that happens in us, it is something we do,” and that it depends on practical knowledge of sensorimotor contingencies (Noë 2004, p. 1). Kevin O’Regan and Noë describe seeing as “a way of exploring the environment” governed by law-like relations between movement and changes in sensory input (O’Regan and Noë 2001, p. 970). Susan Hurley stresses the “dynamic sensorimotor loop” linking perception and action (Hurley 1998, pp. 401–405). Daniel Hutto and Erik Myin defend a “radically enactive” view, in which basic cognition is constituted by embodied interactions rather than representations (Hutto and Myin 2013, pp. 5–7). Andy Clark’s work on action-oriented representation also emphasizes that the brain is geared toward “rolling engagement with the world” from an egocentric point of view (Clark 1997, pp. 46–51; 2013, pp. 24–27).

3 For discussion of perceptual constancies in nonvisual modalities, see J. Vliegen, T. Van Grootel, and A. Van Opstal, ‘Dynamic Sound Localization during Rapid Eye–Head Gaze Shifts’, Journal of Neuroscience 24 (2004), 9291–9302; M. Taylor-Clarke, P. Jacobsen, and P. Haggard, ‘Keeping the World a Constant Size: Object Constancy in Human Touch’, Nature Neuroscience 7 (2004), 219–220; H. Goerlitz, M. Hübner, and L. Wiegrebe, ‘Comparing Passive and Active Hearing: Spectral Analysis of Transient Sounds in Bats’, Journal of Experimental Biology 211 (2008), 1850–1858; Yoshioka et al. (2007) ‘Perceptual Constancy of Texture Roughness in the Tactile System’; and S. Kuroki, J. Watanabe, K. Mabuchi, S. Tachi, and S. Nishida, ‘Directional Remapping in Tactile Inter-Finger Apparent Motion: A Motion Aftereffect Study’, Experimental Brain Research 212 (2012), 311–320.

4 Gallagher argues that Bermúdez misclassifies proprioception as ordinary perception, overlooking its non-object-oriented, action-linked, and prereflective nature in bodily self-awareness. Rather than presenting the body as an object, proprioception serves as a background awareness of posture and movement, integral to motor control and the minimal self. Gallagher maintains that Bermúdez’s perceptual framing over-intellectualizes proprioception, distorting its phenomenology and its fundamental role in agency (Gallagher 2005, pp. 131–160).

5 Zahavi has addressed related objections in a series of subsequent publications, including his exchanges with Kriegel (2016), his contribution to the “thin, thinner” debate (2017), his paper in Review of Philosophy and Psychology (2018), and his discussions in later edited volumes (e.g. Henriksen et al., 2019; Kriegel, 2020). While this later work refines and clarifies the minimal self-thesis, it does not resolve the central difficulty identified here. Rather, the trajectory of these replies involves a progressive thinning of the notion of for-me-ness. This strategy preserves internal coherence but at the cost of explanatory constraint, leaving the underlying methodological problem unchanged.

6 Recent work by Susanna Schellenberg (2025) emphasizes the polysemy of the first-person concept and argues that minimal forms of subject-involvement are more fundamental than full-fledged self-consciousness. I am sympathetic to this diagnosis. However, the present account does not rely on semantic distinctions among uses of “I.” Instead, it offers a more fine-grained explanatory framework grounded in cognitive architecture. The egocentric-registration plus EGO-file model explains how different forms of subjectivity arise without presupposing any particular theory of first-person concepts. In this respect, the framework does not merely accommodate Schellenberg’s proposal but renders it explanatorily dispensable.
